# Differentially Expressed Genes Related to Flowering Transition between Once- and Continuous-Flowering Roses

**DOI:** 10.3390/biom12010058

**Published:** 2021-12-31

**Authors:** Xingwan Yi, Huabei Gao, Yi Yang, Shumin Yang, Le Luo, Chao Yu, Jia Wang, Tangren Cheng, Qixiang Zhang, Huitang Pan

**Affiliations:** National Engineering Research Center for Floriculture, Beijing Key Laboratory of Ornamental Plants Germplasm Innovation & Molecular Breeding, Beijing Laboratory of Urban and Rural Ecological Environment, College of Landscape Architecture, Beijing Forestry University, Beijing 100083, China; yixingwan@bjfu.edu.cn (X.Y.); 15200060372@163.com (H.G.); jiayouziyi@126.com (Y.Y.); yangshumin@bjfu.edu.cn (S.Y.); luolebjfu@163.com (L.L.); yuchao@bjfu.edu.cn (C.Y.); 13910229248@163.com (J.W.); chengtangren@163.com (T.C.); zqx@bjfu.edu.cn (Q.Z.)

**Keywords:** rose, continuous flowering, flowering transition, phytohormone, WGCNA

## Abstract

Roses are the most important cut flower crops and widely used woody ornamental plants in gardens throughout the world, and they are model plants for studying the continuous-flowering trait of woody plants. To analyze the molecular regulation mechanism of continuous flowering, comparative transcriptome data of once- and continuous-flowering roses in our previous study were used to conduct weighted gene co-expression network analysis (WGCNA) to obtain the candidate genes related to flowering transitions. The expression patterns of candidate genes at different developmental stages between *Rosa chinensis* “Old Blush” (continuous-flowering cultivar) and *R.* “Huan Die” (once-flowering cultivar) were investigated, and the relationship of the key gene with the endogenous hormone was analyzed. The results showed that the expression trends of *VIN3-LIKE 1* (*VIL1*), *FRIGIDA- LIKE 3* (*FRI3*), *APETALA 2- LIKE* (*AP2-like*) and *CONSTANS-LIKE 2* (*CO-like 2*) genes were significantly different between “Old Blush” and “Huan Die”, and the expression trends of *SUPPRESSOR OF OVEREXPRESSION OF CONSTANS1* (*SOC1*) and *CO-like 2* were consistent in the flowering transition of “Old Blush” under different environments. The changes in cytokinin and gibberellic acid (GA_3_) content were different in the two rose cultivars. The overall change trend of the abscisic acid and GA_3_ in the flowering transition of “Old Blush” under different environments was consistent. The promoter sequence of *CO-like 2* contained a P-box element associated with gibberellin response, as well as binding sites for transcription factors. In a word, we found *CO-like 2* associated with continuous flowering and some factors that may synergistically regulate continuous flowering. The results provided a reference for elucidating the molecular regulatory mechanisms of continuous-flowering traits in roses.

## 1. Introduction

Flowering transition is one of the most important developmental processes of higher plants, which is controlled by endogenous and external environmental signals. As for the mechanism of plant flowering transition, the main regulatory pathways have been identified, including the photoperiod pathway, the vernalization pathway, the gibberellin pathway, the autonomous pathway, the aging-dependent pathway, and the temperature pathway [1,2]. In addition, some other endogenous factors, such as glucose metabolism and integrators of flowering, can also affect flowering transition [1,2]. In fact, many studies have shown that the *CONSTANS* (*CO*) and *FLOWERING LOCUS T*(*FT*) genes are the core regulatory factors in the photoperiod pathway [3,4,5], and the *CO-FT* regulation mode is conservative in many species [6]. The CCT domain of the *CO* gene can bind to two CO responsive elements (COREs) at the proximal end of the *FT* promoter to promote its expression [7]. DELLA proteins are important factors in sensing the gibberellin signal. Gibberellins are able to ubiquitinate DELLA proteins, allowing them to be easily degraded [8]. DELLA proteins can also interact with CO to inhibit *CO* transcription, by which the gibberellin pathway can cooperate with the photoperiod pathway to regulate flowering [9]. In the vernalization pathway, the *FRIGIDA* (*FRI*) gene is suggested to play an important role in whether vernalization is required [10]. Vernalization is also known to suppress the expression level of *FRI* to promote flowering [11]. Moreover, *FLOWERING LOCUS C* (*FLC*) can delay flowering by inhibiting the expression of downstream factors such as *FT*, *FD*, and *SUPPRESSOR OF OVEREXPRESSION OF CONSTANS1* (*SOC1*) [10,12,13].

Additionally, the *VERNALIZATION INSENSITIVE 3* (*VIN3*) gene has been proven to inhibit *FLC* [14,15]. Two important factors for temperature sensing are *FLOWERING LOCUS M* (*FLM*) and *SHORT VEGETATIVE PHASE (SVP)* genes. In addition, microRNAs have been found to be important in temperature sensing in plants. The *microRNA156* (*miR156*) can combine with its target gene *SQUAMOSA PROMOTER BINDING PROTEIN-LIKE 3* (*SPL3*) to silence its epigenetic [16]. However, *miR156* and its target gene *SPL*, and *microRNA172* (*miR172*) and its target gene *APETALA2* (*AP2*), are also key regulators in the aging-dependent pathway. *miR172* and *miR156* antagonize each other and jointly regulate the flowering transition of plants with increasing plant age. The autonomous pathway is an intrinsic floral induction pathway independent of others, mainly including *FLOWERING CONTROL LOCUS A* (*FCA*), *FLOWERING LOCUS Y* (*FY*), *FLOWERING LOCUS PA* (*FPA*), *FLOWERING LOCUS K* (*FLK*), *FLOWERING LOCUS D* (*FLD*), *FLOWERING LOCUS VE* (*FVE*), and *RELATIVE OF EARLY FLOWERING 6* (*REF6*). These genes promote flowering by inhibiting *FLC*, while they do not interact with each other [1]. Sugar is the product of photosynthesis and is important in inducing flower development, in addition to serving as energy. The results show that the T6P pathway also affects the flowering transition, while the absence of *TREHALOSE-6-PHOSPHATE SYNTHASE 1* (*TPS1*) delays the flowering of *Arabidopsis thaliana* [17]. The key floral integrators include *FT*, *SOC1*, *LEAFY* (*LFY*), and *APETALA1* (*AP1*), while *FT* is regulated by a series of upstream genes from six pathways. All genes in the floral regulatory network coordinate with each other and collectively regulate the flowering transition.

However, when plants are undergoing a flowering transition, there are many genes that affect phytohormone synthesis, or whose expression is regulated by phytohormone, so that hormones also regulate the flowering transition as a signal molecule in plants. Auxin affects flower morphogenesis and flower bud germination. If auxin is absent during plant growth, terminal buds are not able to differentiate into flower buds and form inflorescences [18]. Cytokinin is also an important phytohormone affecting the growth state of the apical meristem, especially in the initial stage of flower induction; this promotes cell division, tissue differentiation, and growth [19]. Gibberellin can regulate the flowering transition and is considered to be the most important phytohormone [20,21]. Gibberellin can not only terminate vegetative growth to induce flowering transition, but also hinder flower formation [22], thus playing a dual role in the process of flower formation. In *Arabidopsis*, abscisic acid inhibits floral transformation by promoting the expression of *FLC* or interacting with the DELLA protein [23]; however, it can also promote the formation of flower buds, thereby also having a dual purpose [24]. The flowering transition of plants is not regulated by a single phytohormone, but by a variety of phytohormones [25].

There are three blooming modes in roses: once-flowering (OF), continuous-flowering (CF), and occasionally re-blooming (OR). The OF rose cultivars bloom only once, in spring, while the CF cultivars can continuously complete flower transition in the growing season, regardless of changes in environmental conditions [26]. The rose has a relatively small genome and is a model plant for studying the continuous flowering of woody plants [27]. Researchers have isolated genes related to flowering transition in roses, of which 26 genes were homologous to the genes in the flowering regulatory network in *Arabidopsis thaliana* [28]. Among these flowering regulatory networks, great progress has been made in the regulation of continuous flowering by the floral integrator *TERMINAL FLOWER 1* (*TFL1*) homologous gene *KSN*. The earliest researchers found that the locus controlling continuous flowering was linked to *RoKSN* that inhibited flowering [26,29]. In CF roses, the *copia*-like retrotransposon insertion in the second intron of *RoKSN* led to failure in the normal expression of *RoKSN* [26]. By overexpression of *RoKSN* in CF roses, CF roses did not bloom after 18 months [30]. In addition, the null allele of *RoKSN*, *RoKSN^null^*, and the allele *RoKSN^LTR^*, which led to occasional re-blooming of the climbing rose mutants, were also found [26,31]. Recently, a new allele, *RoKSN^A181^*, was identified [32]. Roses with the *RoKSN^A181^* allele, which has significantly less expression of *RoKSN* than OF roses, could rebloom and did not possess both alleles of *RoKSN^copia^* or *RoKSN^LTR^* [32]. The discovery of this allele also explained the reason for the recurrent flowering of the CF cultivar of *Rosa rugosa* without the *copia*-like retrotransposon insertion [33].

At present, a large number of studies have proved the relationship between *KSN* and continuous flowering, but there are still many doubts about the mechanism of continuous flowering. It was initially thought that continuous flowering was controlled by a single recessive gene [34], but a large number of roses with the heterozygous genotype *RoKSN^copia^*/*RoKSN^WT^* had different situations of reblooming [32]. Some authors suggested that continuous flowering may be controlled by double recessive loci, and there may be other loci influencing continuous flowering in addition to *KSN* [35]. Moreover, some researchers speculated that continuous flowering was a quantitative–qualitative trait controlled by multiple genes [36]. In fact, two candidate genes, *SPATULA* (*SPT*) and *DELAY OF GERMINATION 1* (*DOG1*), were found [37]. Moreover, recent studies have also found that continuous flowering may be related to epigenetic modification, which leads to the low expression of *KSN* in the heterozygous continuous-flowering *R. rugosa* Purple branch [38].

Therefore, to further explore the molecular mechanism of continuous flowering, the once-flowering cultivar *R.* “Huan Die” and the continuous-flowering cultivar *R. chinensis* “Old Blush” were used as materials, and the transcriptome data obtained in our previous study were used for weighted gene co-expression network analysis (WGCNA) to obtain candidate genes related to flowering transition. The expression patterns of related genes in the two rose cultivars were investigated at different differentiation stages. The related genes and endogenous hormones related to continuous flowering were analyzed. The results will provide new information to elucidate the flowering characteristics of roses.

## 2. Materials and Methods

### 2.1. Plant Materials and Growing Conditions

The once-flowering rose cultivar “Huan Die” (HD) requires a period of chilling stimulation to undergo flower bud differentiation in spring of the flowering year, whereas the continuous-flowering rose cultivar “Old Blush” (YYF) can bloom continuously over the course of a year without cold vernalization [39]. HD and YYF were planted in the nursery of the China National Engineering Research Center for Floriculture (Beijing) (40°17′ N, 116°39′ E). Meanwhile, some plants of YYF were planted in phytotron, at which the light cycle was 16 h/8 h (day/night), the light intensity was 300 μmol·m^−2^·s^−1^, the temperature was 25 °C/20 °C (day/night), and the relative humidity was 60%/40% (day/night).

### 2.2. Identification of Flower Bud Developmental Stage

**Figure 1:**
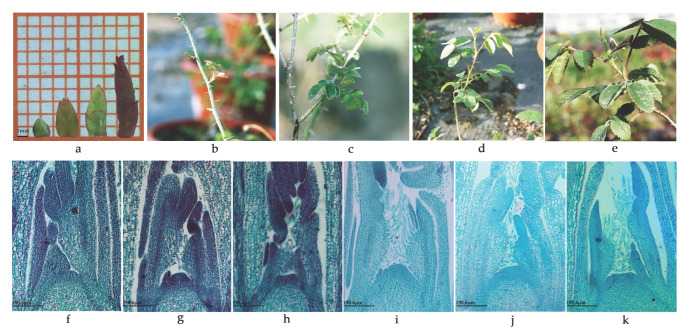
External morphology and internal development status of buds in *Rosa chinensis* “Old Blush” (YYF) and *R*. “Huan Die” (HD): (**a**) external morphology of lateral buds of 1–2 mm, 3–4 mm, 4–5 mm and 6–8 mm in “Old Blush”; (**b**–**e**) shoot apical morphology with 2–3 compound leaves, 5–6 compound leaves, 7–8 compound leaves, and 10–13 compound leaves in “Huan Die”; (**f**–**h**) the internal developmental states of lateral buds of “Old Blush” when bud lengths were 1–2 mm, 3–4 mm, and 4–5 mm, respectively; (**i**–**k**) the internal developmental states of “Huan Die” with expanded 2–3, 5–6, and 7–8 compound leaves, respectively.

To identify flower bud developmental status, 30 plants of “Old Blush” in phytotron were uniformly trimmed at 10 cm high on 10 January 2019, and the first lateral buds were collected to make paraffin sections. According to the length of the lateral bud [40], the lateral buds were divided into four groups (Figure 1a): 1–2 mm (before trimming), 3–4 mm (3 days after trimming), 4–5 mm (5 days after trimming), and 6–8 mm (7 days after trimming). The shoot tips of “Huan Die” were collected according to the number of unfolded compound leaves in newly formed shoots, as follows (Figure 1b–e): 2–3 compound leaves (23 March 2019), 5–6 compound leaves (11 April 2019), 7–8 compound leaves (27 April 2019), and 10–13 compound leaves (17 May 2019). Each sample was collected between 14:00 and 18:00. The buds were quickly soaked in a formalin-acetic acid-alcohol (FAA) solution (formalin, glacial acetic acid, and 50% alcohol in a volume ratio of 5:5:90). After vacuuming, the buds were kept in refrigeration at 4 °C for 2 h. The flower buds were made into permanent paraffin sections with reference to the method of Guo et al. [41]. The paraffin sections were observed under a microscope (Carl Zeiss 444036-9000, Jena, Germany).

### 2.3. WGCNA

WGCNA is an analysis method used to describe the correlation patterns between genes in multiple samples. Genes with similar expression patterns are clustered according to expression level. The method is used to find modules of highly related genes, summarize modules by using the hub genes of modules, and analyze the association between modules or those with specific traits [42]. In a previous study, the transcriptomes of once- and continuous-flowering roses were analyzed using an F1 population of *R. chinensis* “Zhaiye Tengben Yuejihua” (OF) × *R. chinensis* “Old Blush” (CF) as material (accession number in NCBI SRA: SRP12834051-SRP12834062) [43]. In total, 150 F1 seedlings were randomly selected from continuous-flowering seedlings to construct three mixed pools, numbered CF1, CF2, and CF3, with 50 plants in each mixed pool. Using the same method, three mixed pools, numbered IF1, IF2, and IF3, were constructed using 150 once-flowering F1 seedlings. The lateral buds and leaves of each seedling in each mixing pool were sampled separately and mixed evenly. The 4316 differentially expressed genes (DEGs) identified between CF1, CF2, and CF3 and IF1, IF2, and IF3 pools were analyzed by WGCNA in twelve samples in our research. Before WGCNA, cluster analysis was carried out to detect outliers based on the gene expression data in each sample. According to the method of Langfelder and Horvath (2008), R software (v. 4.0.5) and WGCNA (v.1.70.3) software packages were used to construct the gene co-expression correlation matrix and the adjacency function formed by the gene network [42]. The gene hierarchical clustering tree was constructed based on the correlation of gene expression, and the modules were divided according to the clustered relationship among genes. The expression patterns of module genes in each sample were displayed by module eigenvalues, and a heatmap of sample expression patterns was drawn by R software.

### 2.4. Differential Gene Enrichment Analysis

The genes in each module were extracted as prospect genes. Gene Ontology (GO) and Kyoto Encyclopedia of Genes and Genomes (KEGG) Pathway enrichment analysis were performed in the OmicShare tools (www.omicshare.com/tools, accessed on 15 July 2021), using the expression data in buds between CFB and IFB and the KEGG pathway and GO annotation information of the reference genome of *R. chinensis* “Old Blush” (https://www.rosaceae.org/species/rosa/chinensis/genome_v1.0, accessed on 23 November 2021) [31]. The calculated *p*-value passed through FDR correction, taking FDR ≤ 0.05 as a threshold.

### 2.5. Construction of Regulatory Network

Modules related to flowering habits were selected and the genes in the module that ranked in the top 20 with the TOM value (weight) of the key genes were picked out, while the combinations with the TOM value less than 0.20 were filtered out. Then, the node and edge files of the key genes were imported into Cytoscape software (version 3.4.0) to construct a gene co-expression network diagram. Genes with connectivity greater than 23 are the hub genes in the regulatory network.

### 2.6. Analysis of Quantitative Real-Time Polymerase Chain Reaction of Candidate Genes

To explore the expression patterns of genes in “Old Blush” in different environmental conditions, lateral buds of “Old Blush” at four stages were sampled three times (named YYF1, YYF2, and YYF3) in 2019 (Table 1). To contrast the expression differences in genes between different cultivars, the shoot tips at four stages of “Huan Die” were sampled in the natural environment (HD, Table 1). The internal development states of the shoot tips (HD) and lateral buds (YYF) at the four stages were consistent, representing the vegetative growth stage, the early stage of flowering transformation, the stage of flowering transformation, and the late stage of flower development, respectively.

The expression level of genes was measured by quantitative real-time polymerase chain reaction (qRT-PCR), and the *TRANSLATIONALLY CONTROLLED TUMOR PROTEIN* (*TCTP*) gene in the rose was selected as the internal reference gene. The total RNA was extracted by the OMEGA Plant RNA Kit (R6827-01, Omega Bio-Tek Inc., Norcross, Georgia, USA), and the cDNA was obtained by using the TaKaRa PrimeScript RT reagent Kit with gDNA Eraser (RR047A, Takara Bio Inc., Nojihigashi 7-4-38, Kusatsu, Shiga, Japan). The qRT-PCR specific primers were designed according to the online software Primer designing tool (https://www.ncbi.nlm.nih.gov/tools/primer-blast/, accessed on 28 August 2019) (Table S2). The qRT-PCR reaction system was constructed by using a 2 μL cDNA template, 5.5 μL ddH_2_O, 0.5 μL upstream primer, 0.5 μL downstream primer, and 7.5 μL TB Green Primer Ex Taq II (TaKaRa RR420A, Takara Bio Inc., Nojihigashi 7-4-38, Kusatsu, Shiga, Japan). The reaction procedure was as follows: 95 °C, 3 min; 30 cycles (95 °C, 10 s; 60 °C, 30 s). The Mini-option Real-time PCR machine (Bio-Rad, Hercules, CA, USA) was used for qRT-PCR, and each sample was repeated three times. The 2^−ΔΔCt^ method was used to calculate the relative expression of genes.

### 2.7. Determination of Endogenous Hormones

Plant materials used for hormone assays were the same as qRT-PCR materials (Table 1). By the enzyme-linked immunosorbent assay method (ELISA), auxin (IAA), cytokinin (CTK), gibberellin (GA_3_), and abscisic acid (ABA) were measured separately using an IAA ELISA Kit (ml147100, Suzhou Comin Biotechnology Co., Ltd. Suzhou, China), a CTK ELISA Kit (ml026149, Suzhou Comin Biotechnology Co., Ltd. Suzhou, China), a GA_3_ ELISA Kit (ml062451, Suzhou Comin Biotechnology Co., Ltd. Suzhou, China), and an ABA ELISA Kit (ml077235, Suzhou Comin Biotechnology Co., Ltd. Suzhou, China), with three biological replicates for each sample [44].

### 2.8. Promoter Analysis and Prediction of Transcription Factor Binding Sites

The promoter sequence 2kb upstream of the transcriptional start point of the candidate gene was found on the genome website of *R. chinensis* “Old Blush” (https://www.rosaceae.org/species/rosa/chinensis/genome_v1.0, accessed on 23 November 2021) [31]. The biological database Plant CARE (http://bioinformatics.psb.ugent.be/webtools/plantcare/html/, accessed on 23 November 2021) was used to analyze the promoter elements of candidate genes [45]. The co-expressed transcription factors were found by constructing the co-expression network diagram of the genes with the top 350 Tom value (weight) of candidate genes in the module. The transcription factor prediction database JASPAR (http://jaspar.genereg.net/, accessed on 23 November 2021) was used to predict the binding sites of transcription factors and candidate gene promoter regions.

### 2.9. Statistical Analysis

IBM SPSS Statistics 25.0 software was used to analyze the significance of the difference (*p* < 0.05). Univariate analysis of variance was used to analyze the effects of multiple factors on the tested indexes. When the factor was single, the significance of differences was analyzed by one-way ANOVA. When the variance was homogeneous, and Sig. was greater than 0.05, the LSD multiple comparative analysis method was used; in contrast, for multiple comparison the Tamhane’s T2 was used.

## 3. Results

### 3.1. Flower Bud Development of Roses

The external flower bud developmental morphology of “Old Blush” is shown in Figure 1. When lateral bud lengths ranged from 1–2 mm to 6–8 mm, the bud external morphology changed from being tightly coated by bud scales to a subtle opening of the external bud scale; then, the bud further elongated apically to the most peripheral bud scale, and leaf morphology could be identified. As for the internal developmental state, the bud development stage was initially the vegetative growth stage at 1–2 mm before trimming, during which the growth cones bulged upward, and the bud scale and leaf primordium differentiated next to the growth cone (Figure 1f). Then, three days after trimming at 3–4 mm, when the growth cone further widened and became a wide cone, the development stage was the early stage of flowering transformation (Figure 1g). In this stage, the differentiation of leaf primordia increased, the morphology of compound leaves could be observed, and the gap between young leaves and the growth cone became larger. When the length of the bud was 4–5 mm five days after trimming, the top of the growth cone became wider and concaved downward, and obvious convex sepal primordia began to appear on both sides of the growth cone (Figure 1h). At this time, the bud changed from vegetative growth to reproductive growth, in the stage of flowering transformation. After that time, the bud was in the late stage of flower development.

In “Huan Die”, when the newly formed shoots expanded by 2–3 compound leaves on March 23, 2019, the stem tip growth cone was conical, which was in the vegetative growth stage (Figure 1b,i). When the shoots spread by 5–6 compound leaves on April 11, 2019, the growth cone was a wide cone, which was in the early stage of flowering transformation (Figure 1c,j). When the shoots spread by 7–8 compound leaves on April 27, 2019, the growth cone widened and flattened, and was in the flowering transition stage (Figure 1d,k). When the shoots grew 10–13 compound leaves on May 17, 2019, in “Huan Die”, the flower buds could be observed, which were already in the later stage of flower development (Figure 1e).

The flower bud differentiation time of “Old Blush” was significantly shorter than that of “Huan Die”. “Old Blush” could complete the flower transformation in approximately one week and could continuously carry out the flower transformation over a year. This was in contrast to “Huan Die”, which needed to undergo cold vernalization, and underwent floral transition only in spring, which took around two months to complete. However, the micromorphological sign of the transformation from vegetative growth to reproductive growth was the same: i.e., the growth cone varied from an upward convex morphology to a broadening and flattening shape.

### 3.2. Key Modules Related to Flowering Habits Obtained by WGCNA Analysis

**Figure 2:**
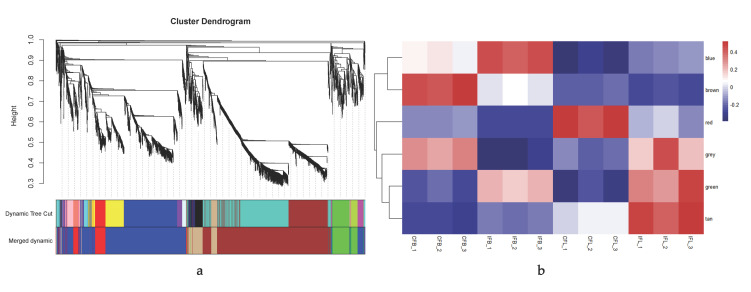
Division of modules and module eigenvalues heatmap: (**a**) clustering dendrogram of genes and division of modules. Merged dynamic: blue, brown, red, grey, green, and tan. These six modules were divided after further merging seventeen modules; (**b**) module eigenvalues heatmap of six regrouped modules: CFB-1, CFL-1: buds and leaves in CF1 pools; CFB-2, CFL-2: buds and leaves in CF2 pools; CFB-3, CFL-3: buds and leaves in CF3 pools; IFB-1, IFL-1: buds and leaves in IF1 pools; IFB-2, IFL-2: buds and leaves in IF2 pools; IFB-3, IFL-3: buds and leaves in IF3 pools.

Cluster analysis of all samples according to gene expression showed that the repeatability of samples was good (Figure S1). Samples of buds and leaves were clustered separately, and the samples of once and continuous rose cultivars were clustered separately. In Figure S2, when R^2^ reaches a plateau or 0.8, the minimum power value is 14, at which the mean connectivity of the genes is approximately 328. Based on all calculated results, the gene modules were divided into 17 groups. As some modules were quite similar in expression, a similarity of 0.75 was chosen to further merge the modules, and the modules were divided into six groups (Figure 2a). Among the six modules, the genes in the blue module were highly expressed in the buds of once-flowering roses, and the gene expression in the grey module was the lowest (Figure 2b). On the contrary, the expression of genes in the brown module was the highest in the buds of continuous-flowering roses, and the expression in the tan module was the lowest. However, the expressions of the tan module in the buds of different blooming modes were both negative and showed little difference, with little correlation with flowering habits. Therefore, the correlation between genes in the blue, brown, and grey modules and flowering habits was a key point of focus in our research.

### 3.3. Hub DEGs Obtained by Enrichment Analysis in Modules

**Figure 3:**
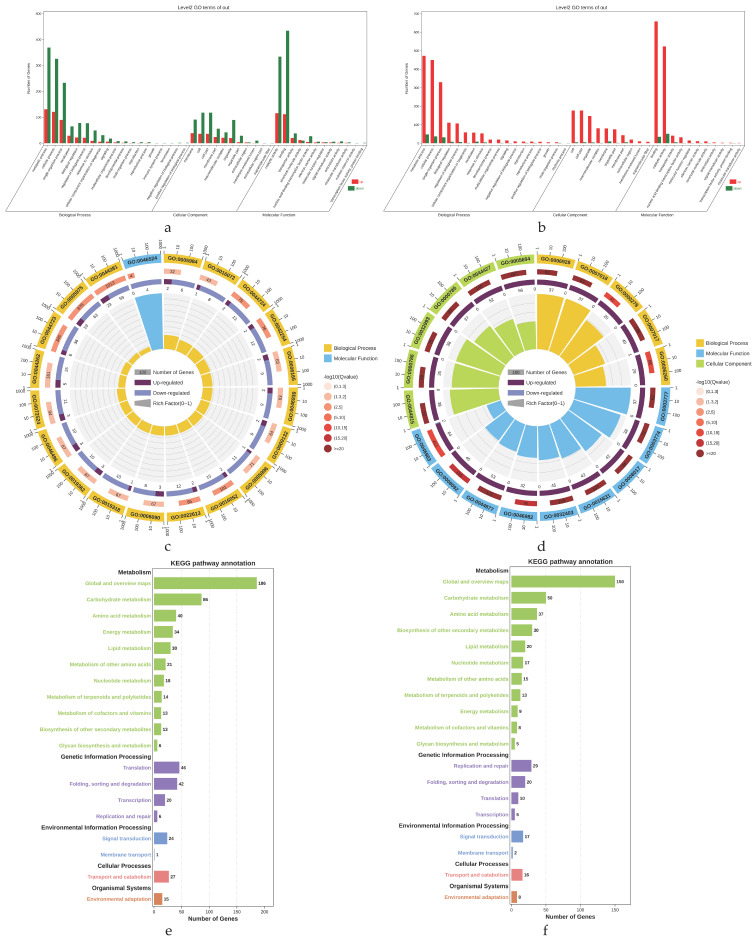
Enrichment analysis of genes in modules: (**a**) secondary classification of GO annotation in blue module; (**b**) secondary classification of GO annotation in brown module; (**c**) GO enrichment analysis circle plot in blue module; (**d**) GO enrichment analysis circle plot in brown module. First circle: top 20 enriched GO terms, outside the circle is the coordinates of the number of genes, and different colors represent different GO categories; second circle: the number and Q value of the genomic background gene in this classification; third circle: the proportion and value of up and downregulated genes; fourth circle: rich factor value of each classification and each cell of background auxiliary line represents 0.1; (**e**) secondary classification of KEGG pathway annotation in blue module; (**f**) secondary classification of KEGG pathway annotation in brown module.

A total of 1615 genes were obtained from the blue module, of which 984 were annotated in three categories of cellular component, molecular function, and biological process in the GO database (Figure 3a). There were 1744 genes in the brown module, of which 1110 genes were annotated in the GO database (Figure 3b). However, because there were only 17 genes in the grey module, enrichment analysis was not carried out. In the category of cellular component, genes within two modules were annotated with a higher proportion of cell and cell parts. In the category of molecular function, genes were mainly distributed in two subcategories, catalytic activity and binding. In the category of biological process, genes were mainly annotated in metabolic process, cellular process, and single-organism process (Figure 3a,b). As can be seen in Figure 3c, there are 20 significantly enriched GO terms in the blue module, which are drawn primarily from the biological process category. The names of the significantly enriched GO terms are shown in Figure S3a, and most of the genes are significantly enriched in GO terms related to metabolic processes, especially the carbohydrate metabolic process, containing GO:0005975, GO:0044723, GO:0044724, GO:0044262, and GO:0016052. On the other hand, the top 20 GO terms are also highly enriched in the brown module (Figure 3d). In the category of molecular function, genes are significantly enriched upon microtubule motor activity and motor activity and significantly enriched upon the movement of cell or subcellular component and microtubule-based movement in the biological process, while they are significantly enriched on the chromosomal part, the DNA packaging complex, and the protein-DNA complex in the cellular component (Figure S3b).

A total of 530 genes in the blue module were annotated to KEGG pathways, and 516 genes in the brown module were annotated (Figure 3e,f). The genes were annotated in five categories (metabolism, genetic information processing, environmental information processing, cellular processes, and organismal systems). Most of the genes within both modules were annotated on the metabolism pathway, in which genes were additionally mainly annotated on global and overview maps and carbohydrate metabolism (Figure 3e,f). Only three KEGG pathways were significantly enriched in the blue module, which contains pathways of carbon metabolism (ko01200), carbon fixation in photosynthetic organisms (ko00710), and glycolysis/gluconeogenesis (ko00010), whereas four pathways were significantly enriched in the brown module containing DNA replication (ko03030), base excision repair (ko03410), starch and sucrose metabolism (ko00500), and homologous recombination (ko03440) (Figure S3c,d).

**Figure 4:**
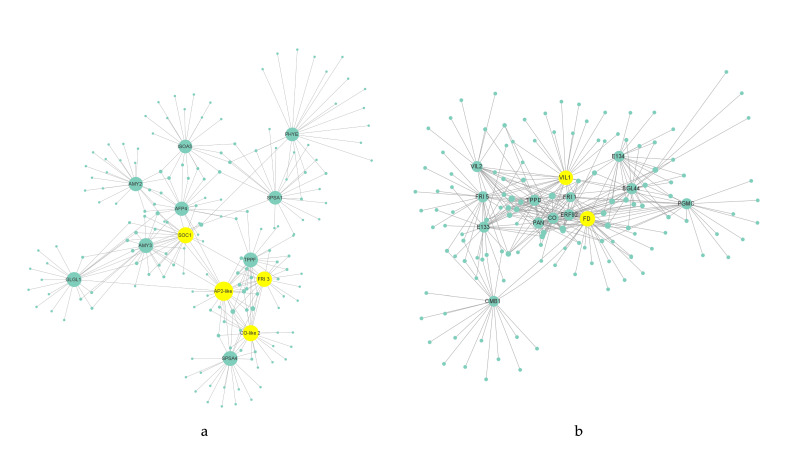
Regulatory network of candidate genes in modules; the yellow circle represents the hub genes in the module: (**a**) gene regulatory network of blue module; (**b**) gene regulatory network of brown module.

We focused on the genes that were significantly enriched or related to the flowering regulation pathways. The annotation information of the related candidate genes is shown in Table S2. The key genes in the blue module included *PHYE* and *CO-like 2* genes in the photoperiod pathway; the *SOC1* gene, which was an integrator of flowering; *AMY3*, *AMY2*, *BAM9*, *SPSA1*, *INV1*, *TPPF*, *GLGL1*, *ISOA3*, *PHS1* and *SPSA4* genes in glucose metabolism pathway; the *FRI 3* gene in the vernalization pathway; the *AP2-like* gene in the aging-dependent pathway; and *AI5L5* and *AFP4* genes in the plant hormone-signaling pathway. The key genes in the brown module included *PGMC*, *ISOA2*, *E133*, *TPPD*, *E134*, *BGL44*, and *BAM1* in the glucose metabolism pathway; *FRI 5*, *VIL2*, *VIL1*, and *FRI 1*, in the vernalization pathway; *CO* in the photoperiod pathway; *ERF92* in the plant hormone-signaling pathway; and *CMB1*, *PAN*, and *FD*, which were integrators of flowering or related to the formation of floral meristem. *SOC1*, *FRI3*, *AP2-like*, and *CO-like 2* genes in the blue module and *VIL1* and *FD* genes in the brown module showed the highest connectivity and the highest correlation with other genes, which were considered to be the hub genes in modules related to flowering transition (Figure 4).

### 3.4. Expression Pattern of Hub DEGs

**Figure 5:**
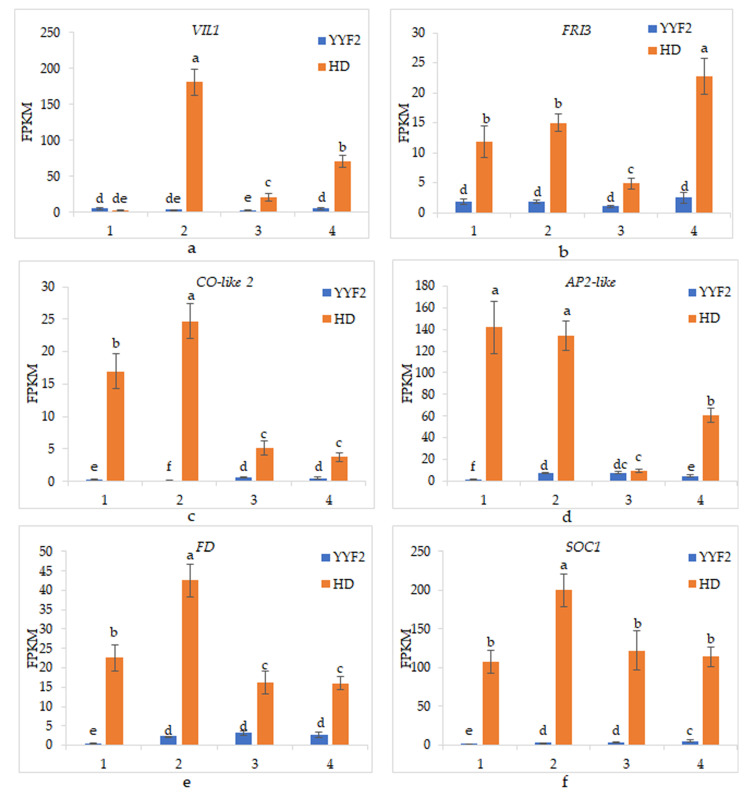
(**a**–**f**) Comparison of gene expression in “Old Blush” and “Huan Die”. The vegetative growth stage, the early stage of flowering transformation, the stage of flowering transformation, and the late stage of flower development are represented by 1, 2, 3, and 4, separately. YYF2 represents “Old Blush” and HD represents “Huan Die”. Error bars represent mean ± SD. The different letters above each column in the figure indicate the significance level of *p* < 0.05.

As shown in Table S3, different cultivar, different flower bud development stages, and different development stages of different cultivars all had a significant impact on gene expression (*p* < 0.05), illustrating that there were significant differences in gene expression in the two cultivars and different stages of flower bud development; thus, these genes were potential candidate genes. The expression levels of these genes in “Huan Die” were all significantly higher than those in “Old Blush” (Figure 5).

The expression of *VIL1* in “Huan Die” showed a significantly increased trend from stage 1 to stage 2, recovered to a lower level in stage 3, and finally increased slightly. In “Old Blush”, the expression of *VIL1* was significantly lower only in stage 3 when compared with other stages (Figure 5a). The *FRI 3* gene did not change significantly before stage 2, then decreased significantly, and finally increased significantly in “Huan Die”. However, there was no significant change trend in “Old Blush” (Figure 5b). The expression trend of *AP2-like* in “Huan Die” was consistent with that of *FRI 3*, while the expression of *AP2-like* in “Old Blush” was up-regulated in stage 2 and down-regulated in stage 4 (Figure 5d). In “Huan Die”, the *CO-like 2* gene was significantly up-regulated in stage 2 and significantly down-regulated afterwards, whereas its expression trend was completely the opposite in “Old Blush” (Figure 5c). The expression trends of these genes in two kinds of cultivars are significantly different, which may be caused by the genes affecting the flowering traits. In “Huan Die”, the expression of *FD* and *SOC1* genes increased significantly from stage 1 to stage 2, decreased in stage 3, and then did not change significantly thereafter. On the other hand, the expression of *FD* and *SOC1* in “Old Blush” was also significantly up-regulated in stage 2, after which there was no significant change, except that *SOC1* was up-regulated in stage 4 (Figure 5e,f). The expression patterns of *FD* and *SOC1* in once-flowering and continuous-flowering rose cultivars remained the same before flowering transformation, meaning that they may not be the key genes.

**Figure 6:**
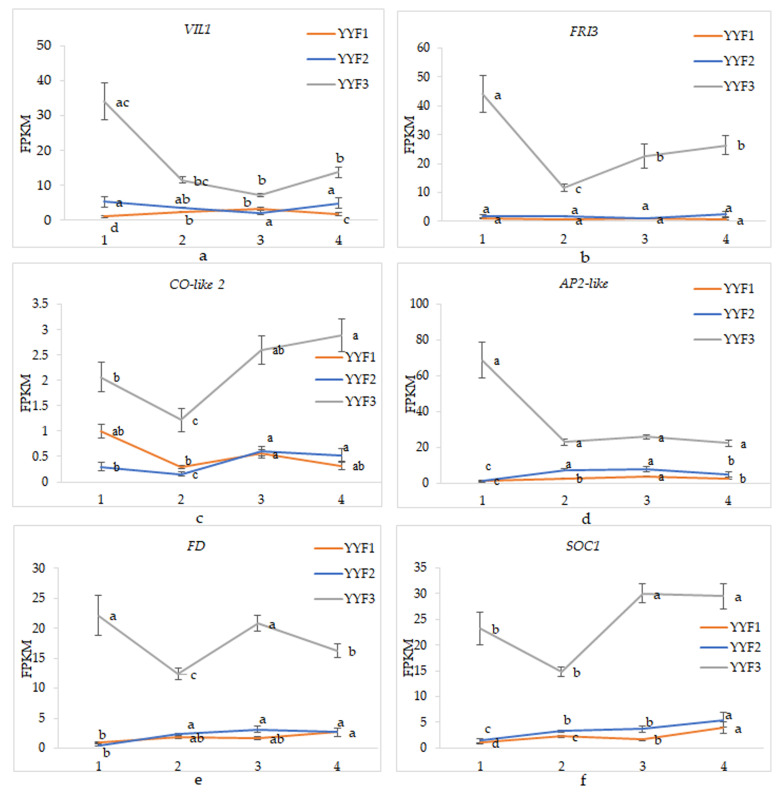
(**a**–**f**) The expression patterns of genes in different environments in “Old Blush”. YYF1, YYF2, and YYF3 represented “Old Blush” under three different environmental conditions. Different flower bud development stages are represented by 1, 2, 3, and 4, separately. Error bars represent mean ± SD. The different letters on each line in the figure indicate the significance level of *p* < 0.05 for that line.

Different environments, and different flower bud development stages in different environments, also had a significant impact on the expression of these genes in “Old Blush” (*p* < 0.05) (Table S4). Among them, the overall expression levels of *VIL1*, *AP2-like*, and *SOC1* genes were significantly different in the three environments (*p* < 0.05). However, the overall expression levels of other genes were significantly different only in the environment of YYF3 (*p* < 0.05). As for the expression trend in the three environments, the overall expression trend of the *SOC1* gene remained upregulated from stage 1 to stage 4 (Figure 6f). Moreover, the expression of *CO-like 2* was down-regulated in stage 2, except in the environment of YYF1, and then up-regulated in stage 3, finally remaining unchanged (Figure 6c). The expression trend of *CO-like 2* remained the same in the three environments. No matter what the environment, the expression trend of these two genes in different environments remained the same, which was similar to the characteristics of “Old Blush”, which itself would continuously bloom independently of environmental influences under a changing external environment. The results illustrated that the genes participated in the regulation process of each flowering transition of “Old Blush”, meaning that these were key genes. However, the expression trends of other genes in three different environments were inconsistent, indicating that their expression trends were greatly affected by the environment (Figure 6a,b,d,e). These results also suggested that the functions of these genes in regulating flowering transition were not uniform in different environments, so they may not be the key genes.

### 3.5. Changing of Endogenous Hormone Content 

**Figure 7:**
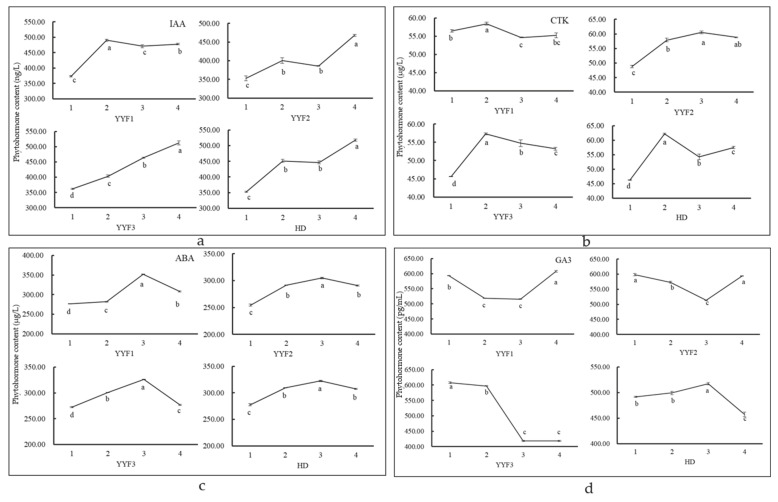
The content of phytohormone in different flower bud differentiation stages: (**a**–**d**) the content of auxin (IAA), cytokinin (CTK), abscisic acid (ABA), and gibberellin (GA_3_) in YYF1, YYF2, YYF3, and HD. Different flower bud development stages are represented by 1, 2, 3, and 4, separately. Error bars represent mean ± SD. The different letters under each line in the figure indicate the significance level of *p* < 0.05.

Under the same environment, between YYF2 and HD, there was the same change trend in IAA and ABA contents (Figure 7a,c). IAA maintained an upward trend and ABA levels continued to rise before stage 3. However, the change trend of CTK was the opposite after stage 2 between YYF2 and HD (Figure 7b). While the concentration of CTK in YYF2 continued to rise after stage 2, the concentration of CTK decreased in HD. Moreover, in “Old Blush”, GA_3_ maintained a downward trend before stage 3, and then increased, which was completely the opposite in “Huan Die” (Figure 7d). On the other hand, the overall change trend of concentrations of ABA and GA_3_ under the three environmental conditions in “Old Blush” was consistent, indicating that they both played a certain role in each flowering transition process and that the role in each process was the same (Figure 7c,d). On the contrary, the content of IAA decreased from stage 2 to stage 3 in YYF1, which was completely the opposite in YYF3 (Figure 7a). Additionally, CTK decreased from stage 2 to stage 3, except in YYF2 (Figure 7b). In a word, GA_3_ showed different change patterns between the OF and CF roses and maintained the same change pattern in “Old Blush” in different environments, which may be an important factor affecting continuous flowering.

### 3.6. Upstream Binding Factors of CO-like 2

The promoter sequence of *CO-like 2* is shown in Figure S4. There were a large number of different types of light-responsive elements such as Box II, G-box, AE-box, Sp1, ACE, Box 4, TCT-motif, and I-box, as well as elements related to stress response, such as the LTR cis-acting element involved in low-temperature responsiveness, the CGTCA-motif and the TGACG-motif cis-acting regulatory elements involved in the MeJA-responsiveness, and the TCA-element involved in salicylic acid responsiveness in the promoter region of *CO-like 2* (Table S5). Moreover, there were cis-acting elements in response to phytohormones, including the auxin-responsive element TGA-element, ABRE cis-acting element involved in the abscisic acid responsiveness, and gibberellin-responsive element P-box. Additionally, the cis-acting regulatory element CAT-box related to meristem expression, and the binding sites of MYB (v-myb avian myeloblastosis viral oncogene homolog) and WRKY transcription factors were also found in the promoter region of the *CO-like 2* gene. The upstream gene is combined with the promoter element of the downstream gene to play a role in regulating the expression of the downstream gene. In the blue module, the transcription factors *WRKY23* of the WRKY family and *PHR1-LIKE 11* (*PHL11*) of the MYB family, with high connectivity with the *CO-like 2* gene, were found (Figure S5), and the binding sites of these genes in the promoter region of the *CO-like 2* gene were recommended for further research (Table S6).

## 4. Discussion

Plants integrate their responses to the external environment and endogenous signals to act on downstream floral integrators, resulting in the transformation from vegetative growth to reproductive growth [46]. These signals include photoperiod, age, temperature, hormones, and carbohydrates [1,17,46]. Through WGCNA and enrichment analysis, some differential genes that may be involved in flowering transformation were found in this study. The differential genes are significantly enriched in the process of carbohydrate metabolism, and these genes play an important role in the process of starch and sucrose metabolism. For example, *AMY3*, *AMY2*, *BAM9*, *BAM1*, *ISOA3*, and *ISOA2* genes are related to starch degradation and metabolism; *SPSA1* and *SPSA4* are related to sucrose metabolism; and *TPPF* and *TPPD* are related to trehalose metabolism. In addition, *FRI* and *VIN3* genes play an important role in the vernalization pathway, and low temperature vernalization affects their expression [11,15]. In the phytohormone signaling pathway, *ERF92*, *AFP4*, and *AI5L5* are involved in the transmission of different phytohormone signals, respectively. In the photoperiod pathway, *PHYE* sense light signals [47], and the expression of *CO* is directly affected by light signals [48]. *AP2* is a key regulator in the aging-dependent pathway. All these signals act on the downstream flowering integrators or flower meristem-related genes *CMB1*, *PAN*, *FD*, and *SOC1* to regulate the flowering transformation of plants.

In the study of the continuous flowering of roses, it is generally believed that *RoKSN*, among the downstream floral integrators, is a key gene. However, many researchers have also posited some doubts and other views regarding the mechanism of continuous flowering [32,35,36,37,38], and more experiments and analyses may be needed to analyze this mechanism. In our study, it was found that *CO-like 2*, *SOC1*, *AP2-like*, *FD*, *VIL1*, and *FRI 3* may be related to flowering habits. In *Arabidopsis*, the *CO-like 2* gene belongs to the CONSTANS family, which contains 16 *CO*-*like* genes, including *CO* that promotes flowering [49]. The CO protein can directly combine with the promoter region of the *FT* gene to promote its expression. Then, the FT protein interacts with the transcription factor FD to form an FT/FD protein complex, which can activate the expression of downstream genes and control the flowering transition [50]. However, overexpression of the *CO-like 2* gene in *Arabidopsis* had little effect on flowering time, because the CO-like 2 protein is different in the amino acid sequence encoded by the first exon compared with the CO protein [51,52]. There is no relevant research or verification on the effect of *CO-like 2* on flowering time in roses. In the vernalization pathway, the *FRI* gene delays flowering by increasing the expression of *FLC* [53]. The FLC protein can bind to the CArG-box domain in *FT* and *SOC1* genes, thereby reducing their transcriptional levels and weakening the impact of the photoperiodic pathway on them [54,55]. *VIN3* encodes a PHD finger protein that deacetylates the histone of FLC chromatin, thereby inhibiting the expression of FLC [14,15]. In addition, *VIN3* can interact with *VERNALIZATION 2* (*VRN2*), and *VERNALIZATION 5* (*VRN5*) to form a conserved protein complex to modify the histone of FLC chromatin [56,57]. In the aging-dependent pathway, with the increase in plant age, the expression level of *miR156* decreases and *SPL* is upregulated, thus promoting flowering [58,59,60]. At the same time, the expression level of *miR172* also increased with age, reducing the translation level of the *AP2* gene to promote flowering [61]. The *SOC1* gene, which encodes MADS-box transcription factors, plays a central role in the flowering regulatory network, is directly regulated by *FT* and binds to the CArG box of the *LFY* promoter to activate its expression [62].

The expression level of these candidate genes in continuous-flowering roses was significantly lower than in once-flowering roses. Previous studies have found that the expression level of *TFL1* in CF roses was significantly lower than in OF roses [63], which has been proved to be due to the insertion of retrotransposon and the null of another allele [26,31]. The reason the expression level of these candidate genes was generally lower in “Old Blush” remains to be further studied. In addition, in our study, it was found that some genes in the flowering regulation network did not show a unified expression pattern in “Old Blush” when the external environmental conditions were different. This phenomenon was found in “Old Blush” in spring, summer, and autumn [41]. However, the continuous-flowering rose can continuously carry out flower transformation despite changes in the external environment. These genes with an inconsistent expression trend may have different functions in regulating flowering in different environments and are not the key factors in controlling continuous flowering. In continuous-flowering roses, there should be key factors that can unify the changes in these genes and control continuous flowering. In our study, the expression pattern of the *CO-like 2* gene was not only significantly different in roses with different flowering habits, but also consistent in continuous-flowering roses in different environments, rendering it the key gene involved in the regulation of continuous flowering. However, whether *CO-like 2* gene regulates the molecular mechanism of continuous flowering requires further verification.

Plant hormones also play an important role in the regulation of the flowering transition. In our study, only GA_3_ showed an opposite change between CF and OF roses, as well as the same law in CF roses under different environments. The content of GA_3_ in “Old Blush” decreased before the flowering transformation, which was consistent with previous studies [41]. GA_3_ could promote the flowering transformation of *Arabidopsis* and inhibit flower formation [22], but exogenous GA_3_ treatment would inhibit the expression of floral integrators *FT*, *SOC1*, and *AP1*, thus affecting the flowering transformation of plants [64,65]. In roses, exogenous GA_3_ application could inhibit flowering in once-flowering cultivars; however, this had no significant effect on continuous-flowering cultivars [66]. As such, the way endogenous gibberellin is involved in the regulation of continuous-flowering rose remains to be studied.

By analyzing the promoter sequence of *CO-like 2* in “Old Blush”, it was found that the expression of the *CO-like 2* gene would be regulated by phytohormones (abscisic acid, gibberellin, and auxin), temperature, light, and transcription factors, which are related to the meristem expression of the plant. In particular, there was a cis-acting element P-box in response to gibberellin. There are other genes in the floral regulatory pathway that can be regulated by gibberellins through promoter regions. In *Arabidopsis*, gibberellin was able to activate the promoter of the *LEAFY* gene through cis-acting elements [67]. Moreover, the gal (the gibberellin signal transduction gene) mutant of *Arabidopsis* failed to bloom because the mutation inhibited the expression of *LFY* [68]. Once gibberellin was applied externally, the expression level of *LFY* in this mutant was upregulated, inducing flowering [69]. However, there are few studies on the promoter sequence of the *CO-like 2* gene. Further research on the interaction between *CO-like 2* and GA_3_ may be necessary to explore whether the *CO-like 2* gene can respond to gibberellin and cooperate with it to regulate the flowering transition. Cis-acting elements located in the promoter region do not encode proteins, and they need specific binding with some transcription factors to play a role in regulating the expression of downstream genes [70]. Most of these regulatory factors can bind to regions within −500 bp of the promoter to activate gene expression [71]. The region within −500 bp of the *CO-like 2* gene promoter contained both MYB binding elements and W-box elements that can be recognized by the WRKY domain; thus, the transcription factors *WRKY23* and *PHL11*, with high connectivity in the module, may be the upstream binding factors regulating *CO-like 2* expression. Via the website, the binding sites were also predicted, which will require verification by means of molecular biology in future research.

## 5. Conclusions

Through WGCNA and gene enrichment analysis in the module, it was found that *CO-like 2*, *SOC1*, *AP2-like*, *FD*, *VIL1*, and *FRI 3* were the hub genes in modules related to continuous flowering. However, only the expression pattern of the *CO-like 2* gene was significantly different in roses with different flowering habits and consistent in CF roses in different environments, which means that the gene may be related to continuous flowering. Additionally, only the change in gibberellin content was different in the two cultivars and the same in CF roses in different environments. The promoter sequence of *CO-like 2* in “Old Blush” contained a P-box element associated with gibberellin response. These results shed new light on elucidating the molecular regulatory mechanism of continuous-flowering roses.

## Figures and Tables

**Table 1:** Plant materials for quantitative real-time polymerase chain reaction and endogenous hormone determination.

Sampling Number	Cultivar	Sampling Time	Plant Tissue	Sampling Stage (bud length in “Old Blush” and Number of Unfolded Compound Leaves in “Huan Die”)				Growth Condition
				1	2	3	4	
YYF1	“Old Blush”	March 15–March 21	lateral bud	1–2 mm	nearly 3 mm	nearly 4 mm	5–6 mm	phytotron
YYF2	“Old Blush”	April 30–May 8	lateral bud	1–2 mm	nearly 3 mm	nearly 4 mm	5–6 mm	open field ^1^
YYF3	“Old Blush”	June 10–June 18	lateral bud	1–2 mm	nearly 3 mm	nearly 4 mm	5–6 mm	open field ^1^
HD	“Huan Die”	March 23–May 17	shoot tip	2–3 unfolded compound leaves	5–6 unfolded compound leaves	7–8 unfolded compound leaves	9–10 unfolded compound leaves	open field ^1^

^1^ The environmental conditions of open field are shown in Table S1.

## Data Availability

The data presented in this study are available in the main text, figures, tables and Supplementary Materials.

## Supplementary Materials

The following are available online at https://www.mdpi.com/article/10.3390/biom12010058/s1, Figure S1: Sample cluster analysis, Figure S2: Analysis of network topology for various soft-thresholding powers, Figure S3: Z-score bubble plot of enrichment analysis, Figure S4: The promoter sequence of *CO-like 2* gene in *Rosa chinensis* “Old Blush”, Figure S5: Regulatory network of *CO-like 2* gene in blue module, Table S1: The natural environmental conditions of the open field in the nursery, Table S2: Candidate gene information and specific primer information of qRT-PCR, Table S3: Tests of between-subjects effects between YYF2 and HD, Table S4: Tests of between-subjects effects in YYF1, YYF2 and YYF3, Table S5: Cis-acting elements and prediction functions of promoter fragments of *CO-like 2* in *Rosa chinensis* “Old Blush”, Table S6: The binding site of transcription factor in the promoter element of *CO-like 2*.
