# Supplementary material for: Differentially Expressed Genes Related to Flowering Transition between Once- and Continuous-Flowering Roses
Source: Biomolecules. 2021 Dec 31;12(1):58. doi: 10.3390/biom12010058 (PMC8773502; doi:10.3390/biom12010058)
Supplement: Supplementary file 1 [file biomolecules-12-00058-s001.zip › Supplementary figure.pdf]

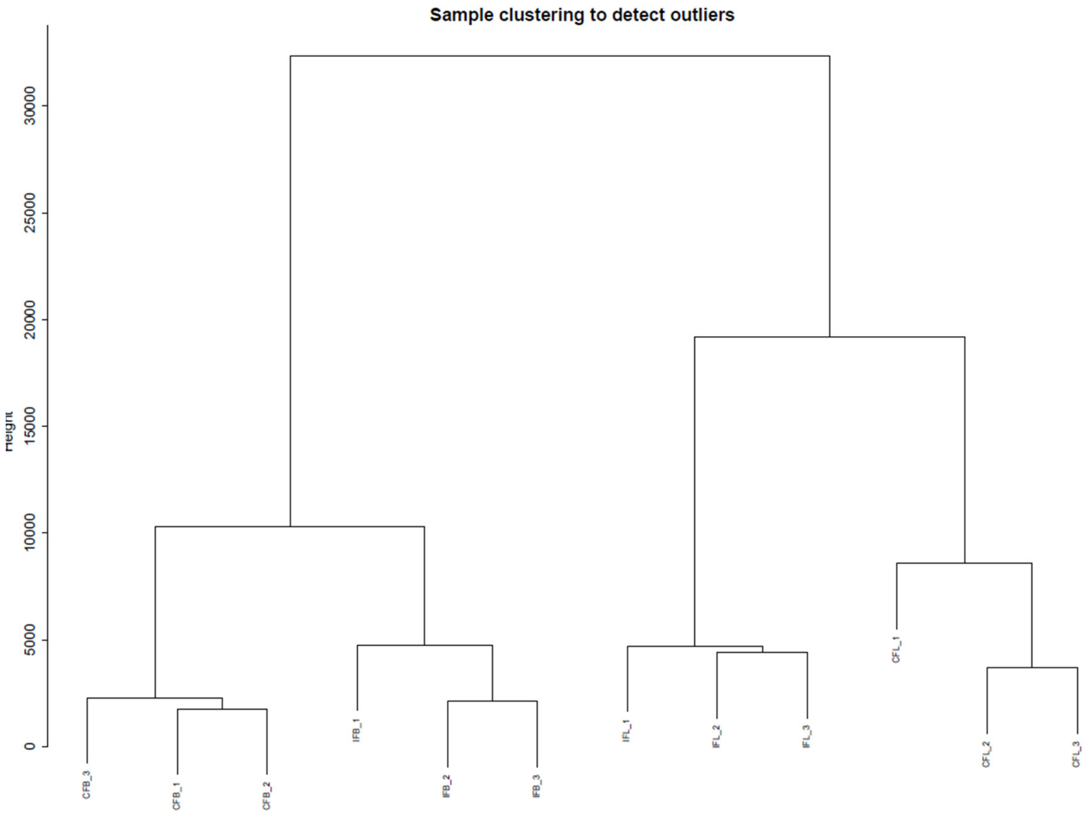

Figure S1: Sample cluster analysis. IFB_1, IFB_2, IFB_3: buds in once flowering rose; CFB_1, CFB_2, CFB_3: buds in continuous flowering rose; IFL_1, IFL_2, IFL_3: leaves in once flowering rose; CFL_1, CFL_2, CFL_3: leaves in continuous flowering rose.

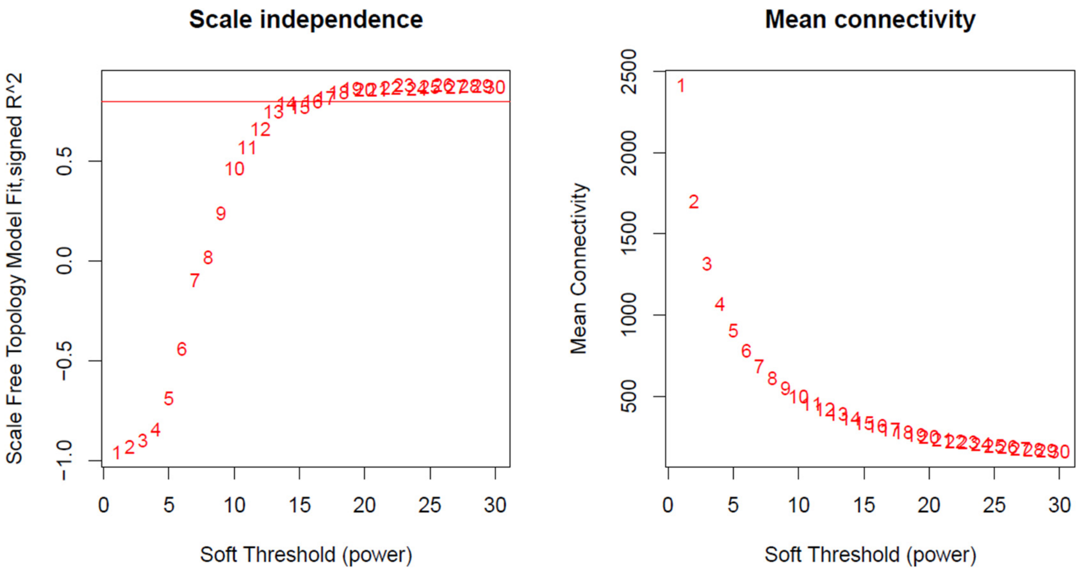

a b

Figure S2. Analysis of network topology for various soft-thresholding powers. (**a**) the curve of Power; (**b**) the curve of mean connectivity.

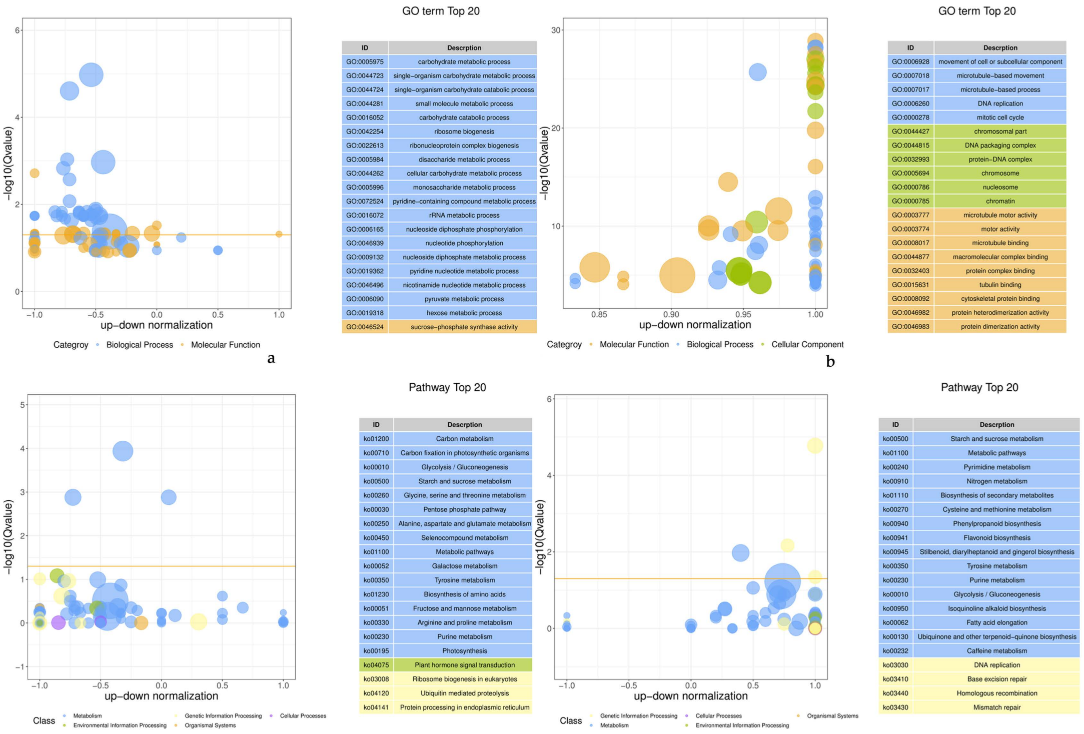

c d

Figure S3. Z-score bubble plot of enrichment analysis. Up-down normalization represents the proportion of the difference between the number of up-regulated genes and the number of down-regulated genes in the total differential genes; The bubble size represents gene count, different colors represent different GO or KEGG categories, and the orange line represents the threshold value of Q-value=0.05; On the right side of every figure is the GO term / Pathway list

of the top 20 Q-value. (a) Go enrichment analysis in blue module; (b) Go enrichment analysis in brown module; (c) KEGG Pathway enrichment analysis in blue module; (d) KEGG Pathway enrichment analysis in brown module.

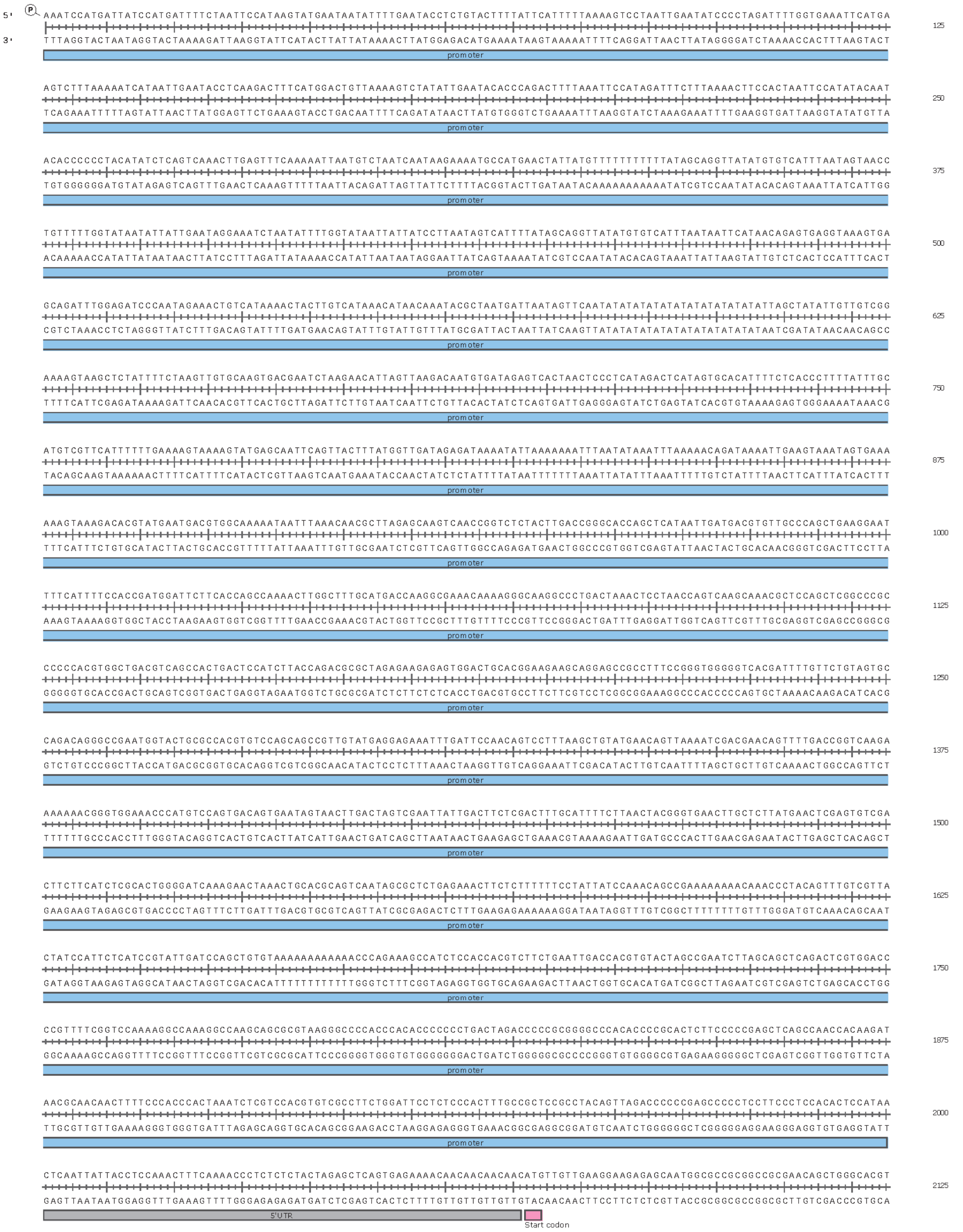

Figure S4. The promoter sequence of *CO-like 2* gene in *Rosa chinensis* 'Old Blush'.

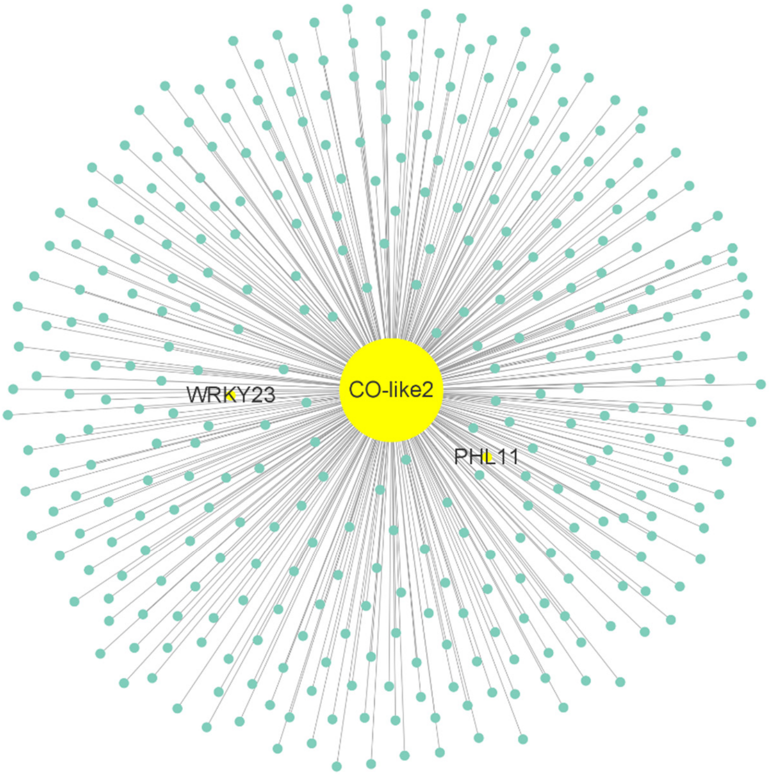

Figure S5. Regulatory network of *CO-like 2* gene in blue module
